# Redetermination of tetra­kis(*N*,*N*-diethyl­dithio­carbamato)tin(IV)

**DOI:** 10.1107/S1600536809018522

**Published:** 2009-05-23

**Authors:** Coco K. Y. A. Okio, Nivaldo L. Speziali

**Affiliations:** a Departamento de Quimíca, Universidad Nacional de Colombia, Sede Bogotá, Bogotá, Colombia; b Departamento de Física, ICEx, UFMG, Brazil

## Abstract

The crystal structure of the title compound, [Sn(C_5_H_10_NS_2_)_4_], was originally determined by Harreld & Schlemper [*Acta Cryst.* (1971), B**27**, 1964–1969] using intensity data estimated from Weissenberg films. In comparison with the previous refinement, the current redetermination reveals anisotropic displacement parameters for all non-H atoms, localization of the H atoms, and higher precision of lattice parameters and inter­atomic distances. The complex features a distorted S_6_ octa­hedral coordination geometry for tin and a *cis* disposition of the monodentate dithio­carbamate ligands.

## Related literature

For the original structure determination, see: Harreld & Schlemper (1971 ▶). For related structures, see: Tiekink (2008 ▶).
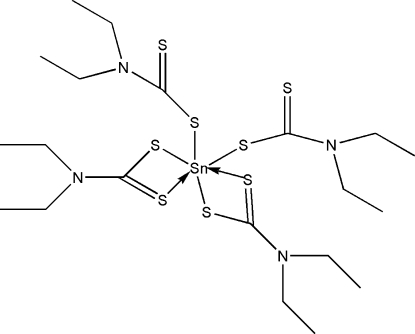


## Experimental

### 

#### Crystal data

[Sn(C_5_H_10_NS_2_)_4_]
*M* *_r_* = 711.73
Monoclinic, 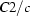

*a* = 16.3250 (2) Å
*b* = 15.7544 (2) Å
*c* = 13.9478 (2) Å
β = 118.995 (2)°
*V* = 3137.64 (8) Å^3^
*Z* = 4
Mo *K*α radiation
μ = 1.36 mm^−1^
*T* = 293 K
0.30 × 0.25 × 0.20 mm

#### Data collection

Oxford Diffraction GEMINI diffractometer
Absorption correction: multi-scan (*CrysAlis RED*; Oxford Diffraction, 2008 ▶) *T*_min_ = 0.794, *T*_max_ = 1.000 (expected range = 0.605–0.761)
64449 measured reflections
8173 independent reflections
6349 reflections with *I* > 2σ(*I*)
*R*_int_ = 0.024

#### Refinement

*R*[*F* ^2^ > 2σ(*F* ^2^)] = 0.037
*wR*(*F* ^2^) = 0.145
*S* = 1.07
8173 reflections
150 parameters
H-atom parameters constrained
Δρ_max_ = 1.24 e Å^−3^
Δρ_min_ = −0.61 e Å^−3^

### 

Data collection: *CrysAlis CCD* (Oxford Diffraction, 2008 ▶); cell refinement: *CrysAlis RED* (Oxford Diffraction, 2008 ▶); data reduction: *CrysAlis RED*; program(s) used to solve structure: *SHELXS97* (Sheldrick, 2008 ▶); program(s) used to refine structure: *SHELXL97* (Sheldrick, 2008 ▶); molecular graphics: *PLATON* (Spek, 2009 ▶); software used to prepare material for publication: *SHELXL97*.

## Supplementary Material

Crystal structure: contains datablocks I, global. DOI: 10.1107/S1600536809018522/bx2209sup1.cif

Structure factors: contains datablocks I. DOI: 10.1107/S1600536809018522/bx2209Isup2.hkl

Additional supplementary materials:  crystallographic information; 3D view; checkCIF report

## Figures and Tables

**Fig. 1.**
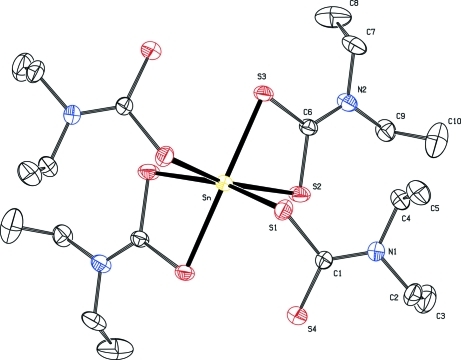
The molecular structure of (I), with the atom-numbering scheme. Displacement ellipsoids are drawn at the 30% probability level. H atoms have been omitted. Unlabeled atoms are related to labeled by the symmetry code (-x+2, y, 1/2-z).

## supplementary crystallographic information

### Comment

Organotin dithiocarbamate compounds continue to attract interest owing to their use as precursors for Chemical Vapor Deposition (CVD) of SnS, as pharmaceuticals, and their structural diversity (Tiekink, 2008). The title compound (I) has been synthesized and its crystal structure reported here. In the previously reported structure (Harreld & Schlemper,1971), no hydrogen atoms were included and the crystal was found to be monoclinic with *Z* = 4, a=15.64 (2) Å, b=15.75 (2) Å, c=13.91 (2) Å, β=112.50 (2)°.These data are slightly different from the new ones due probably to the significantly improved precision with respect to the geometric parameters provided by this redetermination. The molecular structure and the atom-numbering scheme of the title compound are shown in Fig. 1.The tin atom is octahedrally coordinated by two chelating ligands and two monodentate dithiocarbamate ligands with the latter occupying mutually *cis*-positions. Distortions from the ideal octahedral are clearly related to the restricted bite angle of the chelating ligands and further, the asymmetry in the Sn–S bond distances formed by the chelating ligand is related to the *trans* influence exerted by the monodentate ligands, *i.e.* the longer Sn–S bond distance formed by the chelating ligand is *trans*- to the sulfur atom of the monodentate ligand.

### Experimental

Compound (I) was obtained by reacting tin (IV) tetrachloride (883 mg, 3.39 mmol) with sodium *N*,*N*-diethyldithiocarbamate (1160 mg, 6.78 mmol) in ethanol. Orange crystals suitable for X-ray analysis were grown by recrystallization from dichlomethane/hexane.

### Refinement

H atoms were positioned geometrically, with C—H distances in the range 0.96 - 0.97 Å, and constrained to ride on their parent atoms, with *U*_iso_(H) = 1.2–1.5*U*_eq_(C).

### Crystal data

**Table:**

[Sn(C_5_H_10_NS_2_)_4_]	*F*(000) = 1464
*M**_r_* = 711.73	*D*_x_ = 1.507 Mg m^−^^3^
Monoclinic, *C*2/*c*	Mo *K*α radiation, λ = 0.7107 Å
Hall symbol: -C 2yc	Cell parameters from 33587 reflections
*a* = 16.3250 (2) Å	θ = 3.0–37.7°
*b* = 15.7544 (2) Å	µ = 1.36 mm^−^^1^
*c* = 13.9478 (2) Å	*T* = 293 K
β = 118.995 (2)°	Prism, orange
*V* = 3137.64 (8) Å^3^	0.3 × 0.25 × 0.2 mm
*Z* = 4	

### Data collection

**Table:**

Oxford Diffraction GEMINI diffractometer	8173 independent reflections
Radiation source: Enhance (Mo) X-ray Source	6349 reflections with *I* > 2σ(*I*)
graphite	*R*_int_ = 0.024
Detector resolution: 10.4186 pixels mm^-1^	θ_max_ = 37.9°, θ_min_ = 3.0°
π scans	*h* = −27→27
Absorption correction: multi-scan (*CrysAlis RED*; Oxford Diffraction, 2008)	*k* = −27→27
*T*_min_ = 0.794, *T*_max_ = 1.000	*l* = −23→23
64449 measured reflections	

### Refinement

**Table:**

Refinement on *F*^2^	Primary atom site location: structure-invariant direct methods
Least-squares matrix: full	Secondary atom site location: difference Fourier map
*R*[*F*^2^ > 2σ(*F*^2^)] = 0.037	Hydrogen site location: inferred from neighbouring sites
*wR*(*F*^2^) = 0.145	H-atom parameters constrained
*S* = 1.07	*w* = 1/[σ^2^(*F*_o_^2^) + (0.1*P*)^2^] where *P* = (*F*_o_^2^ + 2*F*_c_^2^)/3
8173 reflections	(Δ/σ)_max_ = 0.001
150 parameters	Δρ_max_ = 1.24 e Å^−^^3^
0 restraints	Δρ_min_ = −0.61 e Å^−^^3^

### Special details

**Table:**

Geometry. All e.s.d.'s (except the e.s.d. in the dihedral angle between two l.s. planes) are estimated using the full covariance matrix. The cell e.s.d.'s are taken into account individually in the estimation of e.s.d.'s in distances, angles and torsion angles; correlations between e.s.d.'s in cell parameters are only used when they are defined by crystal symmetry. An approximate (isotropic) treatment of cell e.s.d.'s is used for estimating e.s.d.'s involving l.s. planes.
Refinement. Refinement of *F*^2^ against ALL reflections. The weighted *R*-factor *wR* and goodness of fit *S* are based on *F*^2^, conventional *R*-factors *R* are based on *F*, with *F* set to zero for negative *F*^2^. The threshold expression of *F*^2^ > σ(*F*^2^) is used only for calculating *R*-factors(gt) *etc*. and is not relevant to the choice of reflections for refinement. *R*-factors based on *F*^2^ are statistically about twice as large as those based on *F*, and *R*- factors based on ALL data will be even larger.

### Fractional atomic coordinates and isotropic or equivalent isotropic displacement parameters (Å^2^)

**Table:**

	*x*	*y*	*z*	*U*_iso_*/*U*_eq_	
Sn	1.0000	0.202248 (11)	0.2500	0.03412 (6)	
S4	0.80944 (4)	0.24306 (5)	0.33596 (5)	0.05278 (14)	
S3	1.17240 (3)	0.16968 (3)	0.37176 (4)	0.03869 (10)	
S2	1.01793 (3)	0.09568 (4)	0.39719 (5)	0.04590 (12)	
S1	0.98344 (4)	0.32210 (4)	0.35808 (4)	0.04406 (12)	
C1	0.91736 (14)	0.27902 (13)	0.41621 (15)	0.0378 (3)	
N1	0.95774 (13)	0.28033 (13)	0.52591 (14)	0.0433 (3)	
C2	0.9021 (2)	0.25988 (19)	0.5803 (2)	0.0554 (6)	
H2A	0.8384	0.2794	0.5345	0.066*	
H2B	0.9278	0.2905	0.6492	0.066*	
C3	0.9003 (3)	0.1671 (3)	0.6025 (3)	0.0771 (9)	
H3A	0.8628	0.1579	0.6374	0.116*	
H3B	0.9630	0.1476	0.6497	0.116*	
H3C	0.8740	0.1364	0.5346	0.116*	
C4	1.05786 (18)	0.29665 (16)	0.5989 (2)	0.0528 (6)	
H4A	1.0920	0.2834	0.5601	0.063*	
H4B	1.0802	0.2589	0.6615	0.063*	
C5	1.0788 (2)	0.3865 (2)	0.6392 (2)	0.0725 (8)	
H5A	1.1450	0.3930	0.6861	0.109*	
H5B	1.0466	0.3998	0.6794	0.109*	
H5C	1.0583	0.4243	0.5778	0.109*	
C6	1.13681 (12)	0.10712 (12)	0.44702 (14)	0.0351 (3)	
N2	1.19789 (12)	0.06819 (10)	0.53695 (14)	0.0405 (3)	
C7	1.29923 (14)	0.07507 (16)	0.5782 (2)	0.0543 (6)	
H7A	1.3309	0.0810	0.6574	0.065*	
H7B	1.3121	0.1256	0.5480	0.065*	
C8	1.3367 (3)	−0.0006 (3)	0.5485 (4)	0.0923 (13)	
H8A	1.4028	0.0062	0.5761	0.139*	
H8B	1.3059	−0.0063	0.4702	0.139*	
H8C	1.3256	−0.0505	0.5800	0.139*	
C9	1.16890 (17)	0.01398 (14)	0.60174 (17)	0.0477 (5)	
H9A	1.1044	−0.0033	0.5556	0.057*	
H9B	1.2072	−0.0369	0.6238	0.057*	
C10	1.1771 (4)	0.0561 (3)	0.7004 (3)	0.0904 (12)	
H10A	1.1575	0.0176	0.7388	0.136*	
H10B	1.1380	0.1056	0.6793	0.136*	
H10C	1.2410	0.0723	0.7473	0.136*	

### Atomic displacement parameters (Å^2^)

**Table:**

	*U*^11^	*U*^22^	*U*^33^	*U*^12^	*U*^13^	*U*^23^
Sn	0.02512 (8)	0.04321 (10)	0.03155 (9)	0.000	0.01177 (6)	0.000
S4	0.0354 (2)	0.0764 (4)	0.0425 (2)	−0.0047 (2)	0.01571 (19)	−0.0066 (2)
S3	0.02504 (17)	0.0478 (2)	0.0397 (2)	−0.00161 (15)	0.01292 (15)	0.00968 (17)
S2	0.0283 (2)	0.0557 (3)	0.0517 (3)	−0.00264 (17)	0.01786 (18)	0.0135 (2)
S1	0.0497 (3)	0.0484 (2)	0.0412 (2)	−0.0090 (2)	0.0277 (2)	−0.00716 (19)
C1	0.0360 (8)	0.0449 (8)	0.0333 (7)	0.0028 (7)	0.0175 (6)	−0.0020 (6)
N1	0.0409 (8)	0.0569 (9)	0.0330 (7)	−0.0002 (7)	0.0186 (6)	−0.0033 (6)
C2	0.0600 (14)	0.0738 (16)	0.0439 (10)	−0.0090 (12)	0.0344 (10)	−0.0115 (10)
C3	0.093 (3)	0.086 (2)	0.0622 (17)	−0.0131 (19)	0.0463 (18)	0.0043 (15)
C4	0.0406 (11)	0.0719 (16)	0.0365 (9)	0.0047 (9)	0.0112 (8)	−0.0020 (8)
C5	0.0586 (16)	0.086 (2)	0.0582 (15)	−0.0105 (15)	0.0166 (12)	−0.0127 (14)
C6	0.0277 (6)	0.0383 (7)	0.0355 (7)	−0.0030 (5)	0.0124 (6)	0.0015 (6)
N2	0.0316 (7)	0.0412 (7)	0.0404 (7)	−0.0035 (5)	0.0110 (6)	0.0094 (6)
C7	0.0301 (8)	0.0561 (12)	0.0561 (12)	−0.0041 (8)	0.0047 (8)	0.0155 (10)
C8	0.0567 (18)	0.079 (2)	0.143 (4)	0.0141 (15)	0.049 (2)	0.021 (2)
C9	0.0507 (11)	0.0436 (9)	0.0432 (9)	−0.0068 (8)	0.0183 (8)	0.0093 (7)
C10	0.144 (4)	0.077 (2)	0.0719 (19)	−0.028 (2)	0.069 (2)	−0.0128 (16)

### Geometric parameters (Å, °)

**Table:**

Sn—S1	2.5111 (5)	C4—H4A	0.9700
Sn—S1^i^	2.5111 (5)	C4—H4B	0.9700
Sn—S3^i^	2.5366 (4)	C5—H5A	0.9600
Sn—S3	2.5366 (4)	C5—H5B	0.9600
Sn—S2	2.5567 (5)	C5—H5C	0.9600
Sn—S2^i^	2.5567 (5)	C6—N2	1.316 (2)
S4—C1	1.663 (2)	N2—C7	1.468 (3)
S3—C6	1.7327 (19)	N2—C9	1.478 (3)
S2—C6	1.7247 (18)	C7—C8	1.488 (5)
S1—C1	1.769 (2)	C7—H7A	0.9700
C1—N1	1.341 (2)	C7—H7B	0.9700
N1—C4	1.470 (3)	C8—H8A	0.9600
N1—C2	1.474 (3)	C8—H8B	0.9600
C2—C3	1.497 (5)	C8—H8C	0.9600
C2—H2A	0.9700	C9—C10	1.474 (4)
C2—H2B	0.9700	C9—H9A	0.9700
C3—H3A	0.9600	C9—H9B	0.9700
C3—H3B	0.9600	C10—H10A	0.9600
C3—H3C	0.9600	C10—H10B	0.9600
C4—C5	1.501 (4)	C10—H10C	0.9600
			
S1—Sn—S1^i^	82.48 (3)	N1—C4—H4B	108.9
S1—Sn—S3^i^	98.433 (17)	C5—C4—H4B	108.9
S1^i^—Sn—S3^i^	99.063 (19)	H4A—C4—H4B	107.7
S1—Sn—S3	99.064 (19)	C4—C5—H5A	109.5
S1^i^—Sn—S3	98.434 (17)	C4—C5—H5B	109.5
S3^i^—Sn—S3	156.66 (2)	H5A—C5—H5B	109.5
S1—Sn—S2	90.89 (2)	C4—C5—H5C	109.5
S1^i^—Sn—S2	166.429 (19)	H5A—C5—H5C	109.5
S3^i^—Sn—S2	93.599 (17)	H5B—C5—H5C	109.5
S3—Sn—S2	70.827 (15)	N2—C6—S2	121.35 (14)
S1—Sn—S2^i^	166.427 (19)	N2—C6—S3	121.40 (14)
S1^i^—Sn—S2^i^	90.89 (2)	S2—C6—S3	117.23 (10)
S3^i^—Sn—S2^i^	70.826 (15)	C6—N2—C7	121.91 (17)
S3—Sn—S2^i^	93.599 (17)	C6—N2—C9	122.20 (17)
S2—Sn—S2^i^	97.91 (3)	C7—N2—C9	115.89 (17)
C6—S3—Sn	86.08 (6)	N2—C7—C8	111.7 (2)
C6—S2—Sn	85.61 (6)	N2—C7—H7A	109.3
C1—S1—Sn	104.69 (7)	C8—C7—H7A	109.3
N1—C1—S4	123.21 (16)	N2—C7—H7B	109.3
N1—C1—S1	116.44 (15)	C8—C7—H7B	109.3
S4—C1—S1	120.30 (11)	H7A—C7—H7B	108.0
C1—N1—C4	124.02 (19)	C7—C8—H8A	109.5
C1—N1—C2	119.95 (19)	C7—C8—H8B	109.5
C4—N1—C2	115.94 (19)	H8A—C8—H8B	109.5
N1—C2—C3	113.5 (2)	C7—C8—H8C	109.5
N1—C2—H2A	108.9	H8A—C8—H8C	109.5
C3—C2—H2A	108.9	H8B—C8—H8C	109.5
N1—C2—H2B	108.9	C10—C9—N2	113.6 (2)
C3—C2—H2B	108.9	C10—C9—H9A	108.8
H2A—C2—H2B	107.7	N2—C9—H9A	108.8
C2—C3—H3A	109.5	C10—C9—H9B	108.8
C2—C3—H3B	109.5	N2—C9—H9B	108.8
H3A—C3—H3B	109.5	H9A—C9—H9B	107.7
C2—C3—H3C	109.5	C9—C10—H10A	109.5
H3A—C3—H3C	109.5	C9—C10—H10B	109.5
H3B—C3—H3C	109.5	H10A—C10—H10B	109.5
N1—C4—C5	113.6 (2)	C9—C10—H10C	109.5
N1—C4—H4A	108.9	H10A—C10—H10C	109.5
C5—C4—H4A	108.9	H10B—C10—H10C	109.5

Symmetry codes: (i) −*x*+2, *y*, −*z*+1/2.
